# Experimental pathogenicity of *Skrjabinisakis physeteris* in Wistar rats and its occurrence in *Sarda chiliensis* from Peru: Implications for food safety and zoonotic risk

**DOI:** 10.1016/j.fawpar.2026.e00342

**Published:** 2026-06-05

**Authors:** Araceli Rodriguez-Muñoz, Rosa Martínez-Rojas, Inés Gárate, Carmen Yamashiro, Estrellita Rojas-De-Los-Santos, Abraham Delgado-Escalante, Celso L. Cruces, Jhon D. Chero, Aarón Mondragón-Martínez, Daniel Leonardo Cala-Delgado

**Affiliations:** a Laboratorio de Parasitología en Fauna Silvestre y Zoonosis. Facultad de Ciencias Biológicas, Universidad Nacional Mayor de San Marcos, Peru; b Laboratorio de Parasitología Humana y Animal, Facultad de Ciencias Biológicas, Universidad Nacional Mayor de San Marcos (UNMSM), Lima, Peru; c Departamento Académico de Hidrobiología y Pesquería, Facultad de Ciencias Biológicas, Universidad Nacional Mayor de San Marcos (UNMSM), Lima, Peru; d Instituto de Investigaciones en Ciencias Biomédicas (INICIB), Universidad Ricardo Palma, Santiago de Surco, Peru; e Laboratorio de Zoología de Invertebrados, Departamento Académico de Zoología, Facultad de Ciencias Biológicas, Universidad Nacional Mayor de San Marcos (UNMSM), Peru; f Grupo de Investigación en Ciencias Animales—GRICA, Facultad de Medicina Veterinaria y Zootecnia, Universidad Cooperativa de Colombia, Bucaramanga 680002, Colombia

**Keywords:** *Skrjabinisakis physeteris*, *Sarda chiliensis*, Cox2, Peru, Wistar rats

## Abstract

This study evaluated the experimental infectivity and pathological response of *Skrjabinisakis physeteris* (s.l.) larvae in Wistar rats following exposure to culinary spices commonly used in Peruvian ceviche (*Citrus limon*, *Capsicum pubescens*, *Allium sativum*, and *Piper nigrum*). Between November 2018 and April 2019, 787 specimens of Pacific bonito (*Sarda chiliensis*) were examined, revealing a 49.4% prevalence of anisakid larvae. Morphological analysis showed that 85.6% of recovered larvae corresponded to *Anisakis* Type II, which were selected for experimental infection prior to molecular confirmation. Larvae were exposed to spice treatments for 1 and 3 h to simulate traditional marination practices, and histopathological assessment was performed 48 h post-inoculation. Of the 489 larvae administered, 102 (20.9%) were associated with lesions in gastrointestinal and extraintestinal tissues. Compared with the saline control group, spice-treated larvae showed reduced recovery and pathogenicity, particularly after 3 h of exposure. Molecular characterization of a representative subset of Type II larvae based on mitochondrial *cox2* sequences grouped them with reference sequences of *S. physeteris* available in GenBank. The observed sequence divergence was consistent with intraspecific variation within *S. physeteris* (s.l.). These findings indicate that the infective and pathological responses of *S. physeteris* (s.l.) vary following exposure to culinary spice treatments, while confirming its capacity to induce acute inflammatory and necrotic lesions in a mammalian host. This study provides molecular confirmation of *S. physeteris* (s.l.) in *S. chiliensis* from the central coast of Peru and underscores its potential zoonotic relevance in the context of raw fish consumption. These results contribute to improved risk assessment and seafood safety surveillance in the Southeast Pacific.

## Highlights

• *Skrjabinisakis physeteris* larvae induced acute lesions in Wistar rats.
• Histopathology revealed gastrointestinal and extraintestinal lesions.
• High prevalence of *S. physeteris* was detected in *Sarda chiliensis.*
• Cox2 mtDNA sequencing confirmed *S. physeteris* identification.
• Findings highlight zoonotic risks associated with raw fish consumption.

## 1 Introduction

Anisakid nematodes have complex marine life cycles involving crustaceans as first intermediate hosts, fish and cephalopods as intermediate or paratenic hosts, and marine mammals as definitive hosts. Humans become accidental hosts through the consumption of raw or undercooked fish containing infective third-stage larvae (L3) (Mattiucci and Nascetti, 2008). *Anisakis* larvae are commonly detected in various organs of teleost fish; however, their presence in edible tissues, particularly muscle, raises significant zoonotic concerns (Audicana and Kennedy, 2008). Anisakiasis is a zoonotic disease caused by the accidental ingestion of L3 larvae of *Anisakis* spp. Clinical manifestations include acute gastrointestinal symptoms such as abdominal pain, nausea, vomiting, and diarrhea, as well as allergic reactions ranging from urticaria and angioedema to anaphylactic shock (Audicana and Kennedy, 2008; Mattiucci et al., 2013). In Peru and other South American countries, this parasitosis has been associated with the consumption of traditional raw fish dishes, particularly ceviche (Eiras et al., 2018; Martínez-Rojas et al., 2021; Aco Alburqueque et al., 2020).

Aquatic foods are among the most widely traded commodities worldwide, with global consumption increasing steadily over recent decades (Food and Agriculture Organization of the United Nations (FAO), 2022). In Peru, the Eastern Pacific bonito (*Sarda chiliensis*, Cuvier, 1832) represents a commercially and nutritionally important species. In 2019, national landings reached 92,430 tons, with an average per capita consumption of 3.12 kg, making it one of the most consumed pelagic fish species in the country (Encuesta Nacional de Hogares (ENAHO), 2019). Its widespread use in raw or lightly marinated preparations increases the potential risk of exposure to anisakid larvae.

To mitigate this risk, international sanitary guidelines recommend adequate freezing (−20 °C for ≥24 h) or thermal processing (> 60 °C for ≥1 min) of fishery products prior to consumption (EFSA BIOHAZ Panel, 2010). In Peru, these measures are supported by national sanitary regulations aimed at ensuring the safety of fishery products (Autoridad Nacional de Sanidad e Inocuidad en Pesca y Acuicultura (SANIPES), 2020). However, these measures may not always be strictly applied in traditional culinary practices, which may contribute to the persistence of anisakiasis as a foodborne zoonosis. Cases of *Anisakis* larvae attaching to the gastric mucosa and subsequently removed by endoscopy or expelled orally have been reported both globally and in South America (Baptista-Fernandes et al., 2017; Shimamura et al., 2016; Sohn et al., 2015; Barriga and Salazar, 1999). In addition, recent studies have suggested a potential association between anisakid infection and chronic inflammatory processes, including a possible link with gastrointestinal tumor development (Garcia-Perez et al., 2015; Andreu-Ballester et al., 2024). Despite the increasing number of molecular and ecological studies on anisakid nematodes in the Southeast Pacific, information on their occurrence, species composition, and experimental infective behavior in commercially important fish such as *S. chiliensis* from the central coast of Peru remains limited. In particular, *Skrjabinisakis physeteris* (sensu lato), commonly associated with *Anisakis* Type II larvae, has been less frequently investigated.

Therefore, the aims of the present study were to: (i) determine the prevalence and infection levels of anisakid larvae in *Sarda chiliensis* from the central coast of Peru; (ii) identify the recovered larvae using morphological and molecular approaches; and (iii) evaluate the experimental infective and pathological responses induced by Type II anisakid larvae, later identified as *Skrjabinisakis physeteris* (s.l.) through molecular characterization, following exposure to spice mixtures commonly used in the preparation of Peruvian ceviche in a murine model.

## 2 Materials and methods

### 2.1 Sample collection

From November 2018 to April 2019, 787 specimens of *Sarda chiliensis* were collected (length: 56.50 ± 4.00 cm; weight: 3.15 ± 0.75 kg). The fish were obtained directly from the Ventanilla Fish Market (11°59′22”S, 77°7′35”W) and from biological samples provided by the Instituto del Mar del Perú (IMARPE) (12°04′01”S, 77°09′28”W). All fish were caught off the coast of the Peruvian Sea (Constitutional Province of Callao).

Specimens were examined fresh in the Laboratory of Parasitology in Wildlife and Zoonosis at the Universidad Nacional Mayor de San Marcos. Fish were identified according to Chirichigno and Cornejo (2001).

### 2.2 Larvae collection and treatment with spices

A total of 575 ***Anisakis-*type larvae** were recovered from multiple anatomical sites of **Pacific bonito (***Sarda chiliensis***)**, including the visceral surface, stomach, liver, cecum, spleen, and abdominal skeletal muscle. Fish specimens were initially examined externally and subsequently dissected under controlled laboratory conditions. Larvae were detected by detailed visual inspection and tissue compression under a stereomicroscope, followed by morphological identification to the genus level using light microscopy. According to the morphological criteria of Berland (1961) and the larval classification system of Shiraki (1974), *Anisakis* larvae were assigned to Type I and Type II morphotypes. Type II larvae are characterized by the absence of a mucron and a shorter ventriculus and have been historically associated with *Skrjabinisakis physeteris* (see Murata et al., 2011). All larvae were confirmed to be alive at the time of classification. Only larvae morphologically identified as *Anisakis* Type II were selected for experimental procedures, with species-level identity subsequently confirmed by molecular analysis. Live larvae were placed in sterile glass Petri dishes and exposed to three treatment solutions for 1 or 3 h (see Table 1): (1) *Citrus limon*; (2) *Citrus limon* + *Capsicum pubescens* (5%); and (3) *Citrus limon* + *Allium sativum* (5%) + *Piper nigrum* (2%), while control larvae were maintained in 0.85% saline solution. After exposure, motile larvae were rinsed in physiological saline prior to intragastric administration.

**Table 1:** Effect of spice treatments on S. physeteris larvae in rats. The table shows the details of spice exposure, subgroups, and the number of infected rats across different treatment groups, including the hours of exposure to various spices and the inoculated larvae count.

*S. physeteris* larvae	Treated with spices						No treatment with spices	
Groups	1		2		3		4	
Spices used in the treatment of larvae before infection	*C. limon*		*C. pubescens + C. limon*		*A. sativum* + *C. limon + P. nigrum*		Placebo substance (saline solution)	
Subgroups	A	B	A	B	A	B	A	B
Hours of exposure of larvae on spices	1 h	3 h	1 h	3 h	1 h	3 h	1 h	3 h
N° of infected rats	5	5	5	5	5	5	5	5
Min-Max of larvae inoculated per rat	9–14	10–16	9–12	8–12	10–15	10–15	13–17	10–15
Average number of inoculated larvae	12	12	11	10	13	14	15	12

Spice concentrations (% *w*/*v*) were calculated based on the final volume of the solution using pure lemon juice (*Citrus limon*) as the solvent and vehicle. To ensure replicability and mimic traditional culinary preparation, each ingredient was prepared immediately prior to larval exposure as follows: garlic (*Allium sativum*) was used as finely chopped fresh cloves, rocoto pepper (*Capsicum pubescens*) was used as fresh pulp without seeds, and black pepper (*Piper nigrum*) was added as commercially ground powder. For the lemon juice, only the manually squeezed pulp juice was utilized, completely discarding the peel. The specified proportions of each ingredient were mixed directly in pure lemon juice and manually homogenized immediately before larval exposure, following traditional ceviche preparation procedures. All treatments were performed at room temperature (approximately 20–22 °C) under controlled laboratory conditions. The control group was exposed exclusively to sterile saline solution (0.9% NaCl) to maintain the osmotic pressure and ensure the viability of the unexposed larvae during the experimental period.

### 2.3 Experimental study

Forty male Wistar rats, aged 16–20 weeks and weighing between 150 and 180 g, were obtained from the National Institute of Health (INS), Lima, Peru. The animals were housed in ventilated cages with perforated lids under controlled laboratory conditions (temperature 20 ± 2 °C, relative humidity 78 ± 5%, and a 12-h light/dark cycle), with ad libitum access to food and water throughout the experimental period. Type II *Anisakis* larvae were selected for the experiment because this morphotype was predominant in *Sarda chiliensis* and exhibited greater viability and motility compared to Type I larvae (see Table 1). For each treatment group, larvae were exposed to the designated spice preparation for either 1 or 3 h, as detailed in Table 1. Each treatment included 8–17 larvae per rat, with three independent biological replicates. A control group was also included, in which larvae were treated only with 0.9% sodium chloride solution, to serve as a baseline for comparison with the spice-treated groups. Larval viability was verified immediately prior to inoculation by assessing spontaneous movement and response to mechanical stimulation under a stereomicroscope. Only actively motile larvae were used for oral inoculation. Prior to oral inoculation, rats were fasted for 12 to 16 h (with free access to water) to ensure direct contact between the larvae and the gastric mucosa. No sedation was administered prior to the procedure. Intragastric intubation was performed using gentle manual restraint by experienced personnel, and handling time was minimized to reduce animal stress. Larvae were administered into the rats' stomachs by intragastric intubation, following the method of Figueiredo Jr. et al. (2012), using a modified No. 6 nasogastric probe connected to a 10 mL syringe to ensure precise and gentle delivery.

The infection protocol included five Wistar rats per treatment group and exposure time, resulting in subgroups of five animals each. A control group receiving only 0.9% saline solution by intubation was also included to provide reference material for histological comparison. Following inoculation, animals were monitored closely, and necropsies were conducted 48 h post-infection. The 48 h post-inoculation period was selected to evaluate early larval migration and acute tissue responses rather than long-term persistence. Euthanasia was performed using an overdose of sodium pentobarbital (100 mg/kg). Death was confirmed by the absence of spontaneous respiration and heartbeat, loss of corneal and pedal reflexes, and fixed dilated pupils. Cardiac auscultation and observation for at least 2 min were performed to confirm cardiopulmonary arrest before necropsy. During necropsy, the anatomical location and condition (alive or dead) of the larvae were recorded, and tissue lesions were documented histologically (see Table 2). Recovered larvae were assessed for viability immediately upon recovery. Viability criteria were based on (1) spontaneous motility observed under a stereomicroscope, and (2) response to gentle mechanical stimulation with a fine probe. Larvae were placed in a drop of 0.9% saline at approximately 37 °C and observed for up to 2 min; specimens showing active, coordinated movement or reflexive twitching after stimulation were classified as alive, whereas larvae displaying no movement or no response to repeated mechanical stimulation were classified as dead. Larvae recovered free in the abdominal cavity were stored at −20 °C for molecular identification, while those associated with tissue lesions were processed for histopathology. Fragments of larvae embedded within host tissues were also preserved at −20 °C for genetic confirmation. For the purpose of this study, pathogenicity was defined as the ability of larvae to induce histologically detectable tissue lesions following experimental infection.

**Table 2:** Distribution of pathogenic larvae in different organs after spice treatments and control in rats. The table shows the percentage and number of larvae found in various anatomical locations, including the abdominal cavity, stomach, liver, intestines, and abdominal muscles, after different exposure times and treatment conditions.

Treatment time			Pathogenic larvae on average after five replicates								Total pathogenic larvae
	Group	Spices	Abdominal cavity			Stomach	Liver	Intestine		Abdominal muscle	
			Free	Embedded in abdominal fat, greater omentum or mesentery	Cysts in mesentery, greater omentum and abdominal fat			small intestine	large intestine		
1 h	G 1	T1	4.92% (3/61)	3.28% (2/61)	3.28% (2/61)	1.64% (1/61)	1.64% (1/61)	1.64% (1/61)	0.00% (0/61)	0.00% (0/61)	16.39% (10/61)
	G 2	T2	5.56% (3/54)	12.96% (7/54)	0.00% (0/54)	0.00% (0/54)	0.00% (0/54)	1.85% (1/54)	0.00% (0/54)	3.70% (2/54)	24.07% (13/54)
	G 3	T3	4.69% (3/64)	7.81% (5/64)	7.81% (5/64)	0.00% (0/64)	0.00% (0/64)	1.56% (1/64)	0.00% (0/64)	1.56% (1/64)	23.44% (15/64)
	G 4	C	13.33% (10/75)	10.67% (8/75)	1.33% (1/75)	2.67% (2/75)	0.00% (0/75)	0.00% (0/75)	2.67% (2/75)	1.33% (1/75)	32.00% (24/75)
3 h	G 1	T1	5.00% (3/60)	0.00% (0/60)	0.00% (0/60)	1.67% (1/60)	0.00% (0/60)	1.67% (1/60)	0.00% (0/60)	0% (0/60)	8.33% (5/60)
	G 2	T2	2.04% (1/49)	4.08% (2/49)	0.00% (0/49)	2.04% (1/49)	2.04% (1/49)	0.00% (0/49)	0.00% (0/49)	2.04% (1/49)	12.24% (6/49)
	G 3	T3	3.03% (2/66)	3.03% (2/66)	0.00% (0/66)	0.00% (0/66)	0.00% (0/66)	1.52% (1/66)	1.52% (1/66)	1.52% (1/66)	10.61% (7/66)
	G 4	C	10.00% (6/60)	18.33% (11/60)	1.67% (1/60)	3.33% (2/60)	0.00% (0/60)	0.00% (0/60)	1.67% (1/60)	1.67% (1/60)	36.67% (22/60)
Total number of pathogens larvae											20.86% (102/489)

C: Control; T1: *Citrus limon*; T2: *Citrus limon* + *Capsicum pubescens* (5%); T3: *Citrus limon + Allium sativum* (5%) + *Piper nigrum* (2%).

### 2.4 Histological examination

Tissues from the stomach, liver, skeletal muscle, intestine, and mesenteric nodules of experimentally infected rats were fixed in 10% buffered formalin and processed according to standard histological procedures. Briefly, samples were paraffin-embedded, sectioned at 4 μm, and stained with hematoxylin and eosin (H&E) following conventional protocols (Humason, 1979). Histological sections were examined under light microscopy.

### 2.5 Molecular identification

Genomic DNA was extracted from 10 randomly selected larvae recovered from different anatomical sites using a commercial genomic DNA purification kit, following the manufacturer's instructions. The mitochondrial cytochrome *c* oxidase subunit II (cox2) gene was amplified using primers 211F and 210R (Nadler and Hudspeth, 2000), under PCR conditions previously described by Martínez-Rojas et al. (2021), with an annealing temperature of 46 °C. PCR products were visualized on agarose gels, purified, and sequenced bidirectionally by a commercial sequencing service. The obtained sequences were compared with reference sequences available in GenBank using BLAST. All newly generated sequences were deposited in GenBank (see Table 3).

**Table 3:** List of species used in the phylogenetic analyses, with data on the host species, locality and, GenBank accession number for sequences mitochondrial cytochrome *c* oxidase subunit 2 (cox2) gene.

Species	Locality	Host species	Genbank access.	Reference
*Anisakis berlandi*	Japan:Tokyo	*Katsuwonus pelamis*	LC820445	Takano et al., 2024
*Anisakis nascettii*	South-Eastern Atlantic Ocean (off South Africa)	*Mesoplodon mirus*	GQ118164	Mattiucci et al., 2009
*Anisakis nascettii*	Macquarie Island (South Pacific Ocean)	*Moroteuthis ingens*	GQ118173	Mattiucci et al., 2009
*Anisakis pegreffii*	Italy	*Homo sapiens*	JQ900759	Mattiucci et al., 2013
*Anisakis pegreffii*	Peru	*Trachurus symmetricus murphi*	MZ546437	Martínez-Rojas et al., 2021
*Anisakis pegreffii*	Peru	*Scomber japonicus peruanus*	MZ546443	Martínez-Rojas et al., 2021
*Anisakis simplex*	Atlantic Ocean: NE	*Todarodes sagittatus*	MW082624	Cipriani et al., 2021
*Anisakis simplex*	Tunisia: North coast	*Epinephelus marginatus*	MW324555	Bouderbala et al., 2022
*Anisakis simplex*	Denmark: North Sea	*Clupea harengus*	PQ126422	Kumas et al., 2024
*Anisakis simplex*	Denmark: North Sea	*Clupea harengus*	PQ126428	Kumas et al., 2024
*Anisakis simplex*	Denmark: North Sea	*Clupea harengus*	PQ126432	Kumas et al., 2024
*Anisakis typica*	Thailand: The Gulf of Thailand	*Rastrelliger kanagurta*	MZ708828	Chaiphongpachara et al., 2022
*Anisakis typica*	Thailand: The Gulf of Thailand	*Rastrelliger kanagurta*	MZ708832	Chaiphongpachara et al., 2022
*Anisakis ziphidarum*	Japan:Tokyo	*Scomber japonicus peruanus*	AB517573	Suzuki et al., 2010
*Anisakis ziphidarum*	Southeast Atlantic Ocean (South African Coast)	*Mesoplodon layardii*	DQ116430	Valentini et al., 2006
*Anisakis ziphidarum*	Philippine archipelago	*Mesoplodon hotaula*	KF214803	
*Anisakis ziphidarum*	Brazil: Northeast	*Kogia sima*	KP992461	Di Azevedo et al., 2017
*Anisakis ziphidarum*	Island of Korcula: Adriatic Sea		KT822146	Mladineo et al., 2017
*Anisakis ziphidarum*	Mediterranean Sea: the Strait of Sicily and the Strait of Messin	*Diaphus metopoclampus*	KU752205	Gaglio et al., 2018
*Anisakis ziphidarum*	Turkey: Aegean Sea coast	*Scorpaena scrofa*	MZ297326	Aydin and Pekmezci, 2023
*Skrjabinisakis brevispiculata*	Philippine archipelago	*Kogia Sima*	KC342899	Quiazon et al., 2013
*Skrjabinisakis brevispiculata*	Philippine archipelago	*Kogia Sima*	KC342901	Quiazon et al., 2013
*Skrjabinisakis paggiae*	Philippine archipelago	*Kogia Sima*	KC342896	Quiazon et al., 2013
*Skrjabinisakis paggiae*	Philippine archipelago	*Kogia Sima*	KC821730	Quiazon et al., 2013
*Skrjabinisakis physeteris*	Japan:Mie, off Kumano	*Katsuwonus pelamis*	LC543853	Takano et al., 2021
*Skrjabinisakis physeteris*	Japan:Aomori, off Misawa	*Katsuwonus pelamis*	LC820417	Takano et al., 2024
*Skrjabinisakis physeteris*	Japan:Chiba, southeast of Katsuura	*Katsuwonus pelamis*	LC820446	Takano et al., 2024
*Skrjabinisakis physeteris*	Peru	*Scomber japonicus peruanus*	MZ546445	Martínez-Rojas et al., 2021
*Skrjabinisakis physeteris*	Peru	*Scomber japonicus peruanus*	MZ546449	Martínez-Rojas et al., 2021
*Skrjabinisakis physeteris*	Peru	*Scomber japonicus peruanus*	MZ546450	Martínez-Rojas et al., 2021
*Skrjabinisakis physeteris*			MZ972843	
*Skrjabinisakis physeteris*			MZ972862	
*Skrjabinisakis physeteris*	Peru	*Genypterus maculatus*	OR192868	Chero et al., 2023
*Skrjabinisakis physeteris*	Peru	*Genypterus maculatus*	OR192871	Chero et al., 2023
*Skrjabinisakis physeteris*	Peru	*Genypterus maculatus*	OR192873	Chero et al., 2023
*Skrjabinisakis physeteris*	Peru	*Sarda chiliensis*	OR667806	This study
*Skrjabinisakis physeteris*	Peru	*Sarda chiliensis*	OR667807	This study
*Skrjabinisakis physeteris*	Peru	*Sarda chiliensis*	OR667808	This study
*Skrjabinisakis physeteris*	Peru	*Sarda chiliensis*	OR667809	This study
*Skrjabinisakis physeteris*	Peru	*Sarda chiliensis*	OR667810	This study
*Skrjabinisakis physeteris*	Peru	*Sarda chiliensis*	OR667811	This study
*Skrjabinisakis physeteris*	Peru	*Sarda chiliensis*	OR667812	This study
*Skrjabinisakis physeteris*	Peru	*Sarda chiliensis*	OR667813	This study
*Skrjabinisakis physeteris*	Peru	*Sarda chiliensis*	OR667814	This study
*Skrjabinisakis physeteris*	Peru	*Sarda chiliensis*	OR667815	This study
*Ascaris suum*	USA: Illinois	*Sus scrofa domesticus*	MH469672	Camp et al., 2018
*Contracaecum osculatum*	Denmark: Oresund	*Gadus morhua*	OK338701	Karami et al., 2022

### 2.6 Phylogenetic analyses

Phylogenetic analyses were conducted using a codon-aware approach. Sequences of the mitochondrial cox2 gene were aligned using MACSE v2.07 (Ranwez et al., 2018), and poorly aligned positions and regions with excessive gaps were removed using trimAl v1.5.rev1 (Capella-Gutiérrez et al., 2009) with a gap threshold of 0.7, resulting in a final alignment of 531 bp. Phylogenetic reconstruction was performed using IQ-TREE3 v3.1.1 (Nguyen et al., 2015; Minh et al., 2020) under a partitioned scheme by codon position, with the software available at https://github.com/iqtree/iqtree3. The best-fit substitution models for each partition were selected using ModelFinder according to the Bayesian Information Criterion (BIC), with partition merging allowed. Branch support was assessed using 1000 ultrafast bootstrap replicates and SH-aLRT tests.

Bayesian inference (BI) was performed in MrBayes v.3.2.6 (Ronquist et al., 2012) using the same codon-position partition scheme as in the maximum-likelihood analysis. Because some substitution models selected by IQ-TREE are not directly implemented in MrBayes, a GTR + G model was applied to each codon partition, with parameters unlinked across partitions. Two independent runs with four chains each were performed for 5,000,000 generations, sampling every 1000 generations, and discarding the first 25% of trees as burn-in. One species from the genus *Contracaecum* (*Contracaecum osculatum* [Rudolphi, 1802] Baylis, 1920) and *Ascaris suum* were included as outgroups. The results were visualized in FigTree v1.4.4 (Rambaut, 2012).

### 2.7 Statistical analysis

Differences in larval recovery and lesion occurrence among treatment groups were evaluated using Chi-square tests for proportions. When expected frequencies were low, Fisher's exact test was applied. Parasitological parameters, including prevalence (P%), mean intensity (MI), and mean abundance (MA), were calculated using Quantitative Parasitology 3.0 (Reiczigel et al., 2019). Sterne's exact method was used to estimate 95% confidence intervals for prevalence. Mean intensity and mean abundance were compared using bootstrap procedures with 1000 replications to obtain 95% confidence intervals. Statistical significance was accepted at *p* < 0.05.

## 3 Results

### 3.1 Parasitological infection characteristics in *Sarda chiliensis*

A total of 575 anisakid larvae were recovered from 389 of 787 examined specimens of *Sarda chiliensis*, corresponding to an overall prevalence of 49.4% (95% CI: 45.8–53.1%). Mean abundance was 0.73 ± 0.12 larvae per fish, and mean intensity among infected hosts was 1.48 ± 0.21 larvae per infected fish. Larvae were detected primarily in the coelomic cavity, most frequently associated with the visceral surfaces of the stomach (28.2%), liver (21.0%), cecum (18.4%), and spleen (14.8%), while smaller proportions were found on the visceral peritoneum or within the abdominal skeletal muscle (7.6%). Based on morphological criteria, all recovered specimens were assigned to the genus *Anisakis* and classified into two larval morphotypes: Type II (85.6%; 492/575) and Type I (14.4%; 83/575). The infection parameters reported above refer to all anisakid larvae detected. However, only Type II larvae were selected for subsequent molecular characterization and experimental assays. Significant differences in larval distribution among infection sites were detected (Chi-square test, *p* < 0.05), with higher occurrence in the stomach and liver compared to the intestine and spleen.

### 3.2 Experimental infection

Forty-eight h after intragastric administration, 102 out of 489 inoculated larvae (20.9%) were recovered or associated with detectable lesions in host tissues (Table 2). Most larvae were found free in the abdominal cavity or loosely attached to the mesenteric and omental fat (75.5%; 77/102). Smaller proportions were recovered from the stomach (6.9%; 7/102), liver (2.0%; 2/102), small intestine (4.9%; 5/102), large intestine (3.9%; 4/102), and abdominal muscle (6.9%; 7/102). No evidence of granuloma formation was observed at this stage. A significant difference in larval recovery among treatment groups was detected (Chi-square test, *p* = 0.001). No significant differences were observed among the three spice treatments or between exposure times (*p* > 0.05). At 48 h post-inoculation, all recovered larvae were non-viable. Of the 489 inoculated larvae, 387 (79.1%) were not recovered during necropsy, suggesting that a substantial proportion were digested, disintegrated, or otherwise cleared by the host. Prior to inoculation, all larvae exhibited active motility after 1 and 3 h of spice exposure. The recovery rate was higher in the control group (32–36%) compared with spice treated groups (8–24%).

### 3.3 Histopathology of experimental infection

**Fig. 1:**
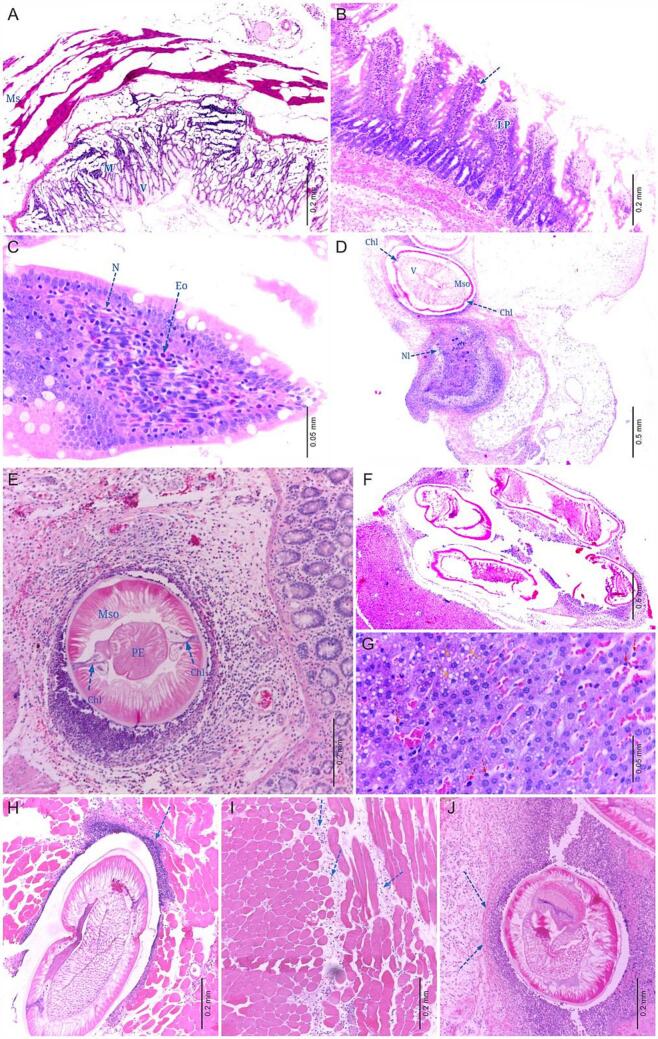
Photomicrographs of tissue sections illustrating pathological changes caused by *S. phytesris* larvae. **1 A.** Muscle layer atrophy in the stomach. The submucosal layer (S), intestinal villi (V), muscle layer (Ms) and mucosal layer (M) are observed. **1B.** Intestinal mucosa. The arrow indicates epithelial atrophy and detachment from the lamina propria (LP). **1C.** Damage to the intestinal villus. Eosinophilic infiltration (Eo) in the lamina propria and necrosis of enterocyte nuclei (N) are observed. **1D.** Bacterial liquefactive necrosis (Nl) adjacent to the parasite. Lateral hypodermal cords (Chl), ventricle (V), and somatic muscle (Mso) are observed. **1E.** Cross-section of a non-pretreated larva at the paraventricular esophagus (PE) level, invading the submucosa. Polymyarian body musculature (Mso) and bilobed hypodermal cords (Chl) surrounded by inflammation are observed. **1F.** Pseudocyst on the surface of the liver. **1G.** Hepatocyte necrosis (red arrow) and fat degeneration (yellow arrow). **1H.** An initial inflammatory response in muscle fibers is observed, with focal eosinophilic myositis and acute parasitic infiltration (blue arrow). **1I.** Multifocal parasitic fibrinoid suppurative myositis in tissue next. **1** **J.** Early tendency for granulomatous formation in the greater omentum. (For interpretation of the references to colour in this figure legend, the reader is referred to the web version of this article.)

Histological examination of rats receiving only 0.9% saline solution revealed normal tissue architecture in the stomach, intestine, liver, skeletal muscle, and mesenteric tissues, with no evidence of inflammatory infiltrates, necrosis, hemorrhage, edema, or granulomatous reactions. Similar histopathological patterns were observed among all larva-inoculated groups, including the untreated larval control group. Histological examination of the stomach revealed focal eosinophilic gastritis. Severe atrophy of the muscular layer was observed (Fig. 1A), along with complete lysis of villi in the mucosal epithelium. Focal hemorrhage and discontinuity of the muscular layer were also present.

The small intestine showed multifocal eosinophilic enteritis. Atrophy of the mucosa, muscular layer, and intestinal villi was evident, together with eosinophilic infiltration in the lamina propria (Fig. 1B). Enterocyte necrosis was observed, characterized by pyknosis, karyorrhexis, and areas of coagulative necrosis (Fig. 1C). A larval nodule was observed on the surface of the small intestine. In the mesenteric gland, areas of liquefactive necrosis associated with bacterial presence were detected (Fig. 1D). In the large intestine (cecum), larvae were observed within the submucosa, associated with colitis characterized by focal suppurative and eosinophilic exudate and diffuse hemorrhage (Fig. 1E). Larvae were also detected in the liver and skeletal muscle. In the liver, specimens were located on the external surface (Glisson's capsule), associated with diffuse eosinophilic hepatitis (Fig. 1F). Adjacent hepatocytes showed coagulative necrosis, fatty degeneration, and diffuse hemorrhage (Fig. 1G). In skeletal muscle, lesions included focal eosinophilic myositis (Fig. 1H) and multifocal fibrinoid suppurative myositis with predominant neutrophilic exudate (Fig. 1I), as well as fibrinoid necrosis, hemorrhage, and intermyofibrillar edema. Extragastrointestinal larval locations included the liver, skeletal muscle, and abdominal cavity. In the abdominal cavity, larvae were observed free, embedded, or forming nodular structures in the mesentery and greater omentum. These nodules were characterized by panniculitis with eosinophilic and suppurative exudate (Fig. 1J). Similar inflammatory patterns were observed surrounding larvae in the stomach and intestine.

### 3.4 Molecular identification and phylogenetic analysis

**Fig. 2:**
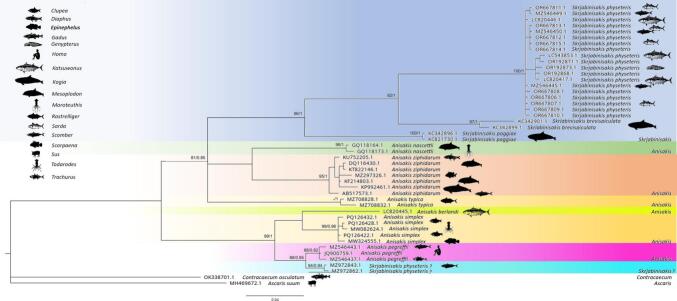
Phylogenetic relationships inferred from maximum likelihood (ML) and Bayesian inference (BI) analyses based on the cox2 dataset. Nodal support is presented as ML/BI; ML values (1000 replicates, only bootstrap values ≥70% are shown) and BI values (only posterior probabilities ≥0.9 are shown). Dashes indicate bootstrap values <70%. *Ascaris suum* and *Contracaecum osculatum* were used as outgroup taxa. The scale bar represents the number of substitutions per site.

Amplification of the mitochondrial cytochrome *c* oxidase subunit II (cox2) gene from 10 larvae produced fragments of approximately 533 bp. Phylogenetic analyses based on cox2 sequences consistently placed all *Anisakis* Type II larvae (*n* = 10) within the *Skrjabinisakis physeteris* clade. Maximum-likelihood and Bayesian inference analyses yielded congruent topologies and provided strong support for the assignment of the recovered larvae to *S. physeteris* (Fig. 2). Within the main *S. physeteris* clade, the analyzed sequences grouped in a supported subclade together with a subset of reference sequences (Fig. 2).

## 4 Discussion

This study documents a high prevalence of *Skrjabinisakis physeteris* (s.l.) larvae in *Sarda chiliensis* from the central coast of Peru, representing the highest infection level reported for this host species in the region. The observed prevalence (49.4%) and mean abundance (0.73 ± 0.12 larvae per fish) exceed previous records from northern Peru (Aco Alburqueque et al., 2020), suggesting spatial variability in infection levels along the Peruvian coast. Such variation may be associated with regional differences in trophic ecology, prey availability, and the distribution of intermediate or paratenic hosts involved in transmission.

The detection of larvae was based on visual inspection and tissue compression, which may underestimate infection levels, particularly in muscle tissues. Previous studies have demonstrated that UV-pressing and artificial digestion techniques provide higher sensitivity than visual examination alone (Levsen et al., 2005; Llarena-Reino et al., 2012). Therefore, the prevalence and abundance values reported here should be interpreted as conservative estimates. Notably, the relatively high infection levels observed despite this methodological limitation reinforce the epidemiological significance of anisakid infection in *Sarda chiliensis* from the central coast of Peru. Although *Anisakis pegreffii* (Type I) has been reported as a dominant anisakid species in Peruvian marine ecosystems (Martínez-Rojas et al., 2021; Chero et al., 2023), the predominance of *S. physeteris* (85.6%) in the present study highlights the relevance of this species in *S. chiliensis.* These findings expand the known host and geographic range of *S. physeteris* in the Southeast Pacific and emphasize the importance of continued surveillance of anisakid assemblages in commercially important fish species.

The sequence divergence observed among the analyzed specimens may reflect phylogenetic structuring within *Skrjabinisakis physeteris* (sensu lato). Previous molecular studies have suggested the existence of distinct phylogenetic lineages within this taxon, including the lineage formerly designated as *Anisakis* sp. 2 (Aco Alburqueque et al., 2020). Although the phylogenetic reconstruction obtained in the present study revealed a supported subdivision within the main *S. physeteris* clade, the resolution provided by a single mitochondrial marker is insufficient to assess species boundaries or taxonomic status. Previous studies have demonstrated that analyses based on a single mitochondrial or nuclear locus may not adequately resolve closely related anisakid taxa or cryptic lineages (Mattiucci and Nascetti, 2006; Takano and Sata, 2022). Therefore, multilocus or mitogenomic approaches will be necessary to clarify the phylogenetic structure and diversity of *S. physeteris* in the Southeast Pacific. For this reason, the specimens analyzed herein are conservatively referred to as *S. physeteris* (s.l.).

Experimental infections in Wistar rats demonstrated that *S. physeteris* larvae are capable of penetrating host tissues and inducing acute lesions within 48 h post-inoculation. Larvae were predominantly recovered from the abdominal cavity and mesenteric tissues, consistent with migratory behavior described in experimental anisakiasis models (Jeon and Kim, 2015; Romero et al., 2014). The absence of granuloma formation at 48 h suggests that the infection remained in an acute phase under the conditions evaluated. These findings provide additional experimental evidence supporting the pathogenic capacity of *Skrjabinisakis physeteris* larvae in a mammalian host, complementing earlier experimental observations (Deardorff et al., 1982; Romero et al., 2014).

Spice-treated groups showed lower larval recovery rates compared with controls, indicating a possible reduction in infective capacity after exposure. However, larvae remained motile prior to inoculation, suggesting that spice treatment did not cause immediate mortality but may have affected subsequent invasive performance. Although the antiparasitic properties of certain plant-derived compounds, such as allicin from garlic (*Allium sativum*), piperine from black pepper (*Piper nigrum*), capsaicinoids from rocoto pepper (*Capsicum pubescens*), and citric acid from lemon (*Citrus limon*), have been reported in vitro, evidence regarding the effects of culinary spice mixtures on anisakid larvae remains limited.

Therefore, the results obtained in this study should be interpreted as exploratory, and further controlled studies are required to elucidate the mechanisms underlying these observations.

It is important to note that the experimental conditions used in this study may not fully replicate natural exposure scenarios. Larvae were exposed directly to spice solutions outside the fish tissue matrix, which may differ from real consumption conditions where larvae are embedded within muscle and ingested with food. Additionally, the murine model employed may not fully reproduce the physiological and immunological responses observed in human anisakiasis. These limitations should be considered when interpreting the experimental findings.

Histopathological findings revealed acute inflammatory responses in gastrointestinal and extragastrointestinal tissues, including eosinophilic infiltration, hemorrhage, and necrosis. These lesions resemble those reported in experimental and clinical anisakiasis caused by *Anisakis simplex* s.s. and *A. pegreffii* (Mattiucci et al., 2017; Baptista-Fernandes et al., 2017), indicating comparable pathogenic potential. Early inflammatory nodules observed in mesenteric tissues may represent initial stages of granulomatous reaction.

Interestingly, the bacterial-associated necrotic lesions observed in some tissues may suggest secondary microbial involvement following larval penetration. Recent hypotheses propose that Anisakis infection and granuloma formation could facilitate bacterial translocation and contribute to subsequent septic processes (Andreu-Ballester et al., 2024). Although bacterial cultures were not performed in the present study, the coexistence of larval invasion and necrotic foci supports the need to further investigate potential parasite–bacteria interactions in experimental anisakiasis models.

The co-occurrence of anisakid species in *S. chiliensis* reflects the ecological complexity of parasite transmission in the Southeast Pacific. Oceanographic variability, including El Niño-associated changes in temperature and prey distribution, may influence parasite dynamics and host exposure patterns. In addition to experimental studies, *S. physeteris* has been implicated in human anisakiasis cases based on morphological identification (Asato et al., 1991). Reports from South America further document its clinical relevance (Cabrera and Suárez-Ognio, 2002; Cabrera, 2010), supporting its recognition as a zoonotic anisakid species. These findings reinforce the public health significance of the high infection levels observed in the present study.

From a food safety perspective, the high infection rate observed in a commercially valuable species commonly consumed in raw preparations underscores the need for strict preventive measures. Thermal processing (> 60 °C for ≥1 min) or freezing at −20 °C for ≥24 h effectively inactivates anisakid larvae (Pardo González et al., 2020; Fioravanti et al., 2021), in accordance with national sanitary guidelines (Autoridad Nacional de Sanidad e Inocuidad en Pesca y Acuicultura (SANIPES), 2020). In conclusion, this study provides experimental evidence that *S. physeteris* can induce acute tissue damage in a mammalian host and documents its high prevalence in *S. chiliensis* from the central coast of Peru. Importantly, the study evaluates these experimental infective and pathological responses following exposure to culinary spice treatments. These findings support the inclusion of *S. physeteris* in regional anisakid surveillance programs and highlight its potential relevance in foodborne zoonotic risk assessments in the Southeast Pacific.

## Animal ethics

All animal experiments and protocols were approved by the Ethical Committee of the Faculty of Medicine at the Universidad Nacional Mayor de San Marcos (Registry Number N°19–0115). The rat experiments were conducted at the Faculty of Biological Sciences, Universidad Nacional Mayor de San Marcos (Permit Numbers B19100394 and R.R. 04692-R-19). The rats were raised under controlled conditions appropriate for the experiment.

## CRediT authorship contribution statement

**Araceli Rodriguez-Muñoz:** Validation, Supervision, Project administration, Methodology, Investigation, Formal analysis, Conceptualization, Writing – review & editing, Writing – original draft. **Rosa Martínez-Rojas:** Validation, Supervision, Software, Project administration, Methodology, Investigation, Writing – review & editing, Writing – original draft. **Inés Gárate:** Validation, Methodology, Investigation, Conceptualization, Writing – review & editing, Writing – original draft. **Carmen Yamashiro:** Validation, Supervision, Methodology, Investigation, Formal analysis, Conceptualization, Writing – review & editing, Writing – original draft. **Estrellita Rojas-De-Los-Santos:** Visualization, Validation, Software, Methodology, Investigation, Writing – review & editing, Writing – original draft. **Abraham Delgado-Escalante:** Validation, Supervision, Methodology, Investigation, Data curation, Conceptualization, Writing – review & editing, Writing – original draft. **Celso L. Cruces:** Software, Methodology, Investigation, Formal analysis, Data curation, Writing – original draft. **Jhon D. Chero:** Software, Methodology, Data curation, Conceptualization, Writing – review & editing, Writing – original draft. **Aarón Mondragón-Martínez:** Supervision, Methodology, Investigation, Formal analysis, Data curation, Conceptualization, Writing – review & editing, Writing – original draft. **Daniel Leonardo Cala-Delgado:** Visualization, Resources, Methodology, Investigation, Funding acquisition, Conceptualization, Writing – review & editing, Writing – original draft. **Daniel Leonardo Cala Delgado:** Writing – review & editing, Writing – original draft.

## Declaration of competing interest

The authors declare that they have no known competing financial interests or personal relationships that could have appeared to influence the work reported in this paper.
